# Stochastic Optimized Relevance Feedback Particle Swarm Optimization for Content Based Image Retrieval

**DOI:** 10.1155/2014/752090

**Published:** 2014-07-09

**Authors:** Muhammad Imran, Rathiah Hashim, Abd Khalid Noor Elaiza, Aun Irtaza

**Affiliations:** ^1^Universiti Tun Hussein Onn Malaysia (UTHM), Parit Raja, 86400 Batu Pahat, Johor, Malaysia; ^2^Universiti Teknologi MARA (UiTM), 40450 Shah Alam, Selangor Darul Ehsan, Malaysia; ^3^University of Engineering and Technology, Taxila, Pakistan

## Abstract

One of the major challenges for the CBIR is to bridge the gap between low level features and high level semantics according to the need of the user. To overcome this gap, relevance feedback (RF) coupled with support vector machine (SVM) has been applied successfully. However, when the feedback sample is small, the performance of the SVM based RF is often poor. To improve the performance of RF, this paper has proposed a new technique, namely, PSO-SVM-RF, which combines SVM based RF with particle swarm optimization (PSO). The aims of this proposed technique are to enhance the performance of SVM based RF and also to minimize the user interaction with the system by minimizing the RF number. The PSO-SVM-RF was tested on the coral photo gallery containing 10908 images. The results obtained from the experiments showed that the proposed PSO-SVM-RF achieved 100% accuracy in 8 feedback iterations for top 10 retrievals and 80% accuracy in 6 iterations for 100 top retrievals. This implies that with PSO-SVM-RF technique high accuracy rate is achieved at a small number of iterations.

## 1. Introduction

In this era of information technology, personal image databases as well as commercial image databases are increasing exponentially. The management of these databases demands effective and efficient content based image retrieval (CBIR) system. Hence, in the past few decades, CBIR has been widely used in various fields such as forensic science, medical science, education, entertainment, and web based image searching. However, there is a gap between low level features and high level semantics, which results in the poor performance of CBIR [1].

For improving the CBIR performance, users can be involved in obtaining feedback during retrieval process which is called relevance feedback (RF). RF is considered as an influential tool [2] and has been applied successfully by many researchers [3–5]. However, to achieve the acceptable level of retrieval accuracy, higher number of feedback iterations is required. This leads to the need for further enhancements in RF.

RF process is performed in two steps as (1) once the retrieved images are displayed, the user labels relevant and irrelevant samples as positive and negative feedback respectively and (2) the CBIR system refines its search procedure based on the labeled feedback samples to improve retrieval performance. These steps are carried out iteratively until the user is satisfied. Although RF process is supported by various techniques, support vector machine (SVM) is a more commonly adopted approach. However, the performance of SVM based RF system is not very effective as the average precision is 0.74 [6] and 0.64 [7]. They consider retrieval process as classification of the feedback obtained from users marking relevant and irrelevant images to construct the training sets. However, these techniques do not conform to the users' desire due to the imbalance set of feedback labeling, where the number of irrelevant feedback outnumbered the number of relevant feedback.

To resolve this problem, this paper has proposed a SVM based CBIR system which is supported by the Particle Swarm Optimization (PSO) algorithm, namely, PSO-SVM-RF. The RF is injected into stochastic optimization process and SVM is trained on the best swarms of PSO algorithm. In the proposed system, PSO is used to minimize the user interaction by evolving the images in relevant set through stochastic method during each iteration based on the user feedback. This results in optimizing the RF process. Based on the PSO output, SVM classifies the relevant and irrelevant images. The use of PSO for SVM also increases the size of training set, especially the size of relevant set. When SVM is trained on the large training set produced by the PSO, it will produce more accurate classification results.

## 2. Background Study

### 2.1. Relevance Feedback

RF is a technique used in information retrieval to collect relevant information from the user. It is effective in enhancing the performance of CBIR. The basic aim of RF is to distinguish between the relevant and irrelevant images displayed by the system [8]. Various researchers have used the different mechanism for RF to improve the performance of CBIR. For example, Koskela et al. [9] proposed PicSOM by using binary RF for the neural network training, which classified the relevant and irrelevant images to improve the performance of CBIR. Bordogna and Pasi [10] also trained the neural network using RF to improve the feedback mechanism for image retrieval. Yap and Wu [11] incorporated Fuzzy logic with RF to retrieve the relevant images. Rota Bulò et al. [12] used graph theory to enhance the performance of RF and introduced the random walker algorithm for improving the speed of RF process. The objective of using graph theory was to minimize the navigational process required in RF process and treat positive and negative images labeled by the user at every RF round as seed nodes for random walker problem. Besides these, various machine learning techniques such as EM, KNN and ANN [13], SVM [14, 15], RBF, and Bayesian inference [16] are also used for enhancing the performance of RF. Among these, SVM is the most popularly used technique which models the retrieval process as classification problem and uses relevant and irrelevant images as training sets [13]. Goh et al. [17] used concept-dependent learning to enhance the RF, where image semantic labels were used to guide the active learning process and SVM was used as base learning algorithm. The combination of active learning with SVM improved the performance of RF substantially. Other than this Djordjevic and Izquierdo [18] used SVM based classifier to improve the performance of RF in achieving better accuracy of relevant image retrieval. SVM ensemble proposed by Yildizer et al. [15] also plays a vital role in RF. However, SVM based RF has some limitations; for example, in case of imbalanced feedback samples it leads to poor results [13]. Similarly Tao et al. [13] have highlighted three problems of SVM based RF techniques. These SVM classifier are become unstable when the size of training set is small, Biasness when positive feedback samples are more less than the negative feedback samples and over fitting that is, when training set is smaller as compared with dimensions of feature vector. Hence, this study has focused on addressing these issues by evolving the training set and increasing the number of relevant images through stochastic nature of PSO. It will enable SVM to have larger relevant set for performing classification task.

### 2.2. Particle Swarm Optimization

PSO is an optimization algorithm proposed by Kennedy and Eberhart. The algorithm simulates the behavior of bird flock flying together in multidimensional space for searching optimum place [19]. The nature of PSO is similar to evolutionary computation such as genetic algorithm (GA). However, swarms in PSO are initialized randomly and then search is carried out for an optimum solution.

The algorithm implements the idea of flying particles in a search space to find the global optimum. PSO algorithm performs its computational process with two models which are the cognition and the social models. Cognition model is used for local search while social model represents the global search. In PSO, flying particles are represented as *P*_*i*_ ∈ [*a*, *b*] where *i* = 1, 2, 3,…, *D* and *a*, *b* ∈ *R*, as *D* is for dimensions and *R* is for real numbers [20]. Each particle contains its own position and velocity, which are initialized randomly. After initialization, the particles search their best positions learning from their own and neighborhood experience. Every particle has to maintain two positions called *p*_best_ and *g*_best_. The *p*_best_ represents the particles' own best position and *g*_best_ is the global best position among all the particles. The position and velocity of each particle are updated based on the following equations:

(1) Vi(t+1)=Vi(t)+C1∗r1(pbest−ni(b))+C2∗r2(gbest−xi(t)),xi(t+1)=xi(t)+Vi(t+1),

where *x*_*i*_ is the position, *V*_*i*_ is the velocity, *p*_best_ is the personal best position, and *g*_best_ is the global best position for PSO. Similarly *r*_1_ and *r*_2_ are two random numbers, their range is [0, 1], and *C*_1_ and *C*_2_ are learning factors representing the cognition and social component, respectively, called acceleration coefficients.

Although PSO has proved its performance for different practical problems [21], however, it stuck into local minima [22]. To overcome this problem, recent research work conducted by the authors has proposed various variants [23–25]. To experience the potential benefits of PSO, it has been applied in several domains such as the classification of the digital contents [26], ad hoc sensor network [27], antennas array designing [28], and neural networks [29]. The PSO has also been explored in the field of CBIR. For example, Chandramouli et al. [30] used PSO to optimize the self-organized features maps (SOM) which is based on the supervised clustering, while Okayama et al. [31] used PSO to improve the retrieval ranking. Wu et al. [32] applied PSO to fine-tune the weights of parameters in similarity computation. To grasp the user semantics, Broilo and De Natale [33] used the PSO as a classifier. On the contrary, this study has applied PSO for improving the performance of CBIR using RF.

## 3. Proposed PSO-SVM-RF Approach

**Figure 1:**
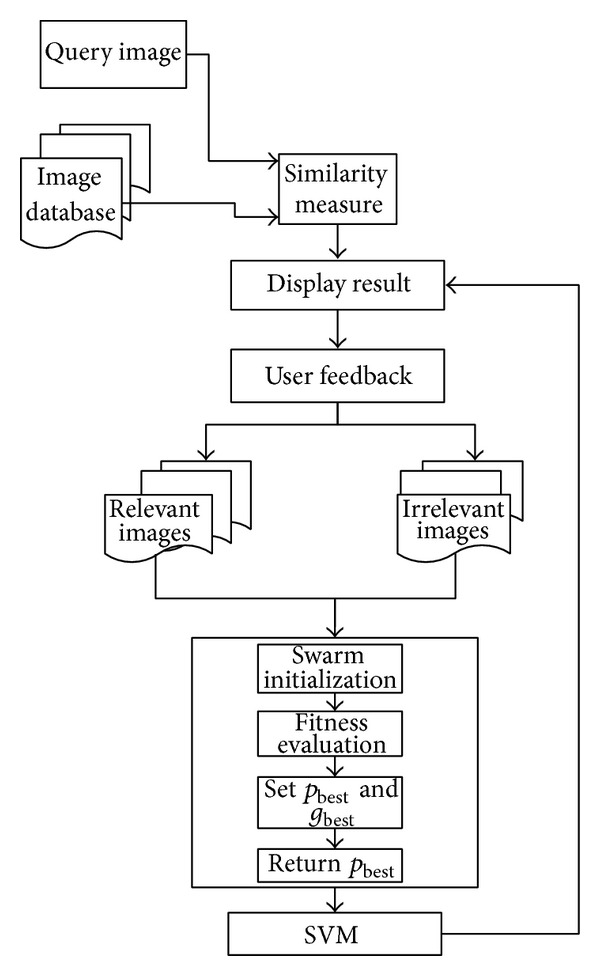
Flow chart of the proposed system.

As mentioned earlier, this study has focused on developing a new approach for enhancing the performance of CBIR using RF by integrating PSO and SVM named as PSO-SVM-RF. It consists of three processes as information gathering from user, swarms updating, and training of SVM. The flow chart of PSO-SVM-RF development is illustrated in Figure 1.

**Figure 2:**
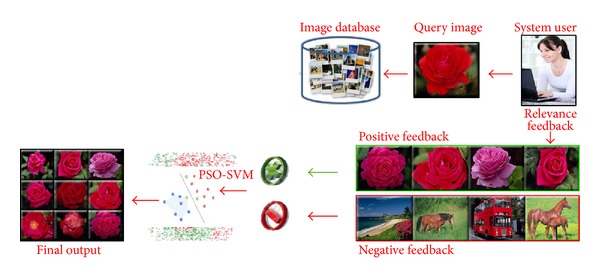
Architecture of the PSO-SVM-RF.

As shown in the Figure 1, PSO-SVM-RF process starts with getting query from the user to search the similar images from the image database. Based on the similarity rank, nearest images known as *N*_FB_ are displayed to the user to obtain user feedback. The similarity between the query image and the database images is measured based on the minimum distance using image feature vector. The distance between the query image and the database images is computed using Manhattan distance which is a similarity measure technique. For displayed images, the user has to mark the relevant images. Subsequently two subsets are created and termed relevant and irrelevant images, where all the images marked by user are treated as relevant images while the rest of the images are labeled as irrelevant images. The relevant images are used to update the swarms of PSO for evolutionary process. The number of relevant images is updated through iterative process. Details of updating the relevant images are discussed in Section 3.3. After the evolutionary process, the output produced by the PSO is used to train the SVM. Finally, SVM will classify relevant and irrelevant images. Then *N*_FB_ nearest images are displayed to the user to collect the first feedback. Architecture of the overall PSO-SVM-RF is described in Figure 2. Detailed mechanism of the PSO-SVM-RF is presented in the following sections.

### 3.1. Swarm Representation

The particles of the PSO are represented by the feature set, and each particle represents a feature vector. The particles fly in the search space available by the features of the image database. To extract the feature set, homogeneous texture descriptors from the MPEG-7 descriptors are used. Homogeneous texture descriptor (HTD) illustrates the directionality, coarseness, and regularity of patterns of an image [34]. HTD consists of the mean, standard deviation, energy, and energy deviation values. Every HTD has a total of 62 feature values. The first two feature values represent the mean and standard deviation of the image, which are calculated as

(2) $$\begin{array}{c} f_{\text{ad}}=\frac{\sum_{x=0}^{w}\sum_{y=0}^{h}f\left(x,y\right)}{n}\\ f_{\mathrm{sd}}=\sqrt{\frac{\sum_{x=0}^{w}\sum_{y=0}^{h}{\left(f(x,y)-f_{\text{ad}}\right)}^{2}}{n}}, \end{array}$$

where *f*(*x*, *y*) is the gray value of image in point (*x*, *y*),* w* and *h* are the width and height of the image, and* n* is the total number of the image pixels.

The remaining 60 feature values of HTD represent energy and energy deviation. Energy and energy deviation are calculated by using frequency layout of the image. The frequency layout is split into 30 channels.

Each of the channels is uniform along the angular direction and nonuniform along the redial direction which follows octave scale.

Overall procedure of extracting 60 feature values from frequency layout consists of three steps which are as follows.

1. Input image *f*(*x*, *y*) is converted into a gray image, where Fourier transform is applied on the gray image to represent it as *F*(*ω*, *θ*) in polar coordinates.
2. Gabor filter is used to strengthen the image information and the results are expressed as *H*_*i*_(*ω*, *θ*), where *i* is the number of feature channels generated by dividing the frequency domain, 1, 2,…, 30.
3. Finally, energy value of each channel is calculated.

Gabor function used in the process for extracting the feature values is described as

(3) $$\begin{array}{c} G_{s,r}\left(w,\theta \right)=e^{\left(-{\left(w-w_{s}\right)}^{2}/2\sigma_{s}^{2})+(-{\left(\theta -\theta \right)}^{2}/2\sigma_{r}^{2}\right)}, \end{array}$$

where *G*_*s*_, *r*(*w*, *θ*) is the point value in the polar coordinates, *w*_*s*_ and *θ*_*r*_ are the radius frequency and angular frequency in feature channels, and *σ*_*s*_ and sigma_*r*_ are the standard deviation of the radius and angle.

For each channel, the center frequencies in the angular and radial directions are divided as

(4) $$\begin{array}{c} \theta_{r}=30^{{}^{\circ}}*r, \end{array}$$

where *r* is the angle index *r* ∈ [0,1, 2,3, 4,5] and each feature channel is 30°. The radius frequency *w*_*s*_ is given as

(5) $$\begin{array}{c} w_{s}=w_{0}*2^{-s},\;s\in \left[0,1,2,3,4\right], \end{array}$$

where *s* is the radius index. The highest radius frequency is marked as 3/4. The standard deviation *σ*_*s*_ and sigma_*r*_ are expressed in the following way:

(6) $$\begin{array}{c} \sigma_{s}=\frac{B_{s}}{\sqrt{2\ln2}},\;\;\sigma_{r}=\frac{B_{r}}{\sqrt{2\ln2}}, \end{array}$$

where *B*_*S*_ and *B*_*r*_ are the spacing value of the radius and angle component.

The energy *e*_*i*_ and energy deviation *d*_*i*_ for the *i*th channel are

(7) ei=log⁡[1+pi],  di=log⁡[1+qi], where  pi=∑w=0+1∑ θ=0°360°Isr2,qi=∑w=0+ 1∑θ=0°360°(Isr2−pi)2,Isr=Gsr(w,θ)·|w|·F(w,θ),

where |*w*| is the Jacobian between the polar and Cartesian coordinates. The final feature vector is as

(8) $$\begin{array}{c} \text{HTD}=\left[f_{ad},f_{sd},e_{1},e_{2},e_{3},\ldots ,e_{3}0,d_{1},d_{2},d_{3},\ldots ,d_{3}0\right]. \end{array}$$

### 3.2. Calculating Distance and User Feedback

To calculate distance between the query image and the database images, each image is described in terms of its features represented by the function *x*_*i*_ = [*x*_*i*_^HTD^]. HTD is calculated using (8) as explained in the earlier section. The calculation of the feature set is usually performed offline for image database. When user selects the query image, it is mapped as *x*_*q*_ in the feature space. From the whole database, the most similar images are displayed based on the distance calculated according to

(9) $$\begin{array}{c} \text{Dist}\left(x_{q}:x_{j}\right)=\text{MNHT}\left(x_{q}^{\text{htd}}:x_{j}^{\text{htd}}\right), \end{array}$$

where MNHT is the Manhattan distance calculated between the query image and the image from the database.

After calculating the Dist(*x*_*q*_ : *x*_*j*_), *j* = 1,2,…, *N*_DB_ (where *N*_DB_ represents the number of images in the database), the result is sorted and presented to the user for collecting feedback. The user then has to mark the images as relevant or irrelevant. Based on the user feedback, the images are categorized into two subsets, namely, relevant *X*_REL_^*k*^ and irrelevant *X*_IRR_^*k*^. It is an iterative process and the size of relevant and irrelevant set is changed after each iteration.

### 3.3. Swarm Initialization and Fitness Evaluation

The swarm initialization process depends on the set of relevant images. The swarms are initialized according to the ranking obtained during first iteration of RF. Every particle *p*_*n*_ is defined as feature vector and the total number of particles (*P*) is expressed as *N*_FB_ ≤ *P* < *N*_DB_. For each particle, random speed vector *v*_*n*_^*k*^, *n* = 1,2,…, *P*, is generated independently for stochastic exploration. The output of the optimization process depends on the fitness function which needs to be minimized or maximized. In this study fitness function is adopted from Broilo and De Natale [33] and defined as follows:

(10) $$\begin{array}{c} \psi_{\left(p_{n}\right)}^{k}=\frac{1}{N_{\mathrm{rel}}^{k}}\overset{N_{\mathrm{rel}}^{k}}{\underset{r=1}{\sum }}\text{Dist}\left(p_{n}^{k};x_{r}^{k}\right)+\frac{1}{\left(1/N_{\text{irr}}^{k}\right)\sum_{i=1}^{N_{\text{irr}}^{k}}\text{Dist}\left(p_{n}^{k};x_{i}^{k}\right)}, \end{array}$$

where *x*_*r*_^*k*^, *r* = 1,2,…, *N*_*rel*⁡_^*k*^, and *x*_*i*_^*k*^, *i* = 1,2,…, *N*_irr_^*k*^, represent the relevant and irrelevant subsets of images. If the particle is near the relevant set, the function *ψ*_*pn*_^*k*^ will produce lower fitness value and vice versa. Lower fitness value indicates that the position of the particle is better. Based on the fitness value, it is easy to reorder the swarms to get the new ranking. In every iteration, the fitness function is varying due to dynamic change in *X*_*rel*⁡_^*k*^ and *X*_irr_^*k*^. Due to this reason, some relevant images can move to the irrelevant zone. During the iterative process, irrelevant images can dominate the relevant images. Hence, in developing the objective function, this limitation was addressed by making fitness function dependent on the inverse of the distance from the irrelevant images. Thus, if the average distance of the particle from the irrelevant images increases, the fitness depends only on the relevant images.

### 3.4. Swarms Evolution and Termination Criteria

After the initialization, swarm evolution process is started. To evolve the swarms, personal best *l*_*n*_^*k*^ of each particle and a global best *g*_*n*_^*k*^ among the entire particles must be defined. In this paper, the personal best and global best are selected and updated with different approach as compared to standard PSO. The query image in PSO-SVM-RF is selected as global best and is updated as an image of the relevant set. The relevant image is chosen based on

(11) $$\begin{array}{c} g_{\text{best}}^{k}=\text{MIN}\left\{\overset{X_{\mathrm{rel}}^{k}}{\underset{j=1}{\sum }}\text{Dist}\left(x_{r};x_{j}\right)\right\};\;x_{r},x_{j}\in x_{\mathrm{rel}}^{K}. \end{array}$$

For every relevant image, the sum of the distances from the other relevant images is computed where the image having shortest distance is selected as *g*_best_. In case of zero relevant images other than the query image, query image is considered as *g*_best_. At the start of swarm evolution process, *p*_best_ is different for each particle as it is initialized by the original feature vector. Updating of the *p*_best_ depends on the result of (10). If *ψ*_(*p*_*n*_)_^*k*^ ≤ *ψ*_(*p*_*n*_)_^*k*−1^, then *p*_best_ will be updated. Depending on the local and global best, speed vector of each particle is updated as

(12) $$\begin{array}{c} v_{n}^{k}=\omega \cdot v_{n}^{k-1}+C_{1}r_{1}\left\{l_{n}^{k}-p_{n}^{k-1}\right\}+C_{2}r_{2}\left\{g^{k}-p_{n}^{k-1}\right\}, \end{array}$$

where *r*_1_ and *r*_2_ are two random numbers, their range is chosen from [0, 1], and *ω* is the inertia weight which is kept static at 0.4. *C*_1_ and *C*_2_ are constants aiming at accelerating the particles towards the cognition related position (i.e., personal best) or social related position (i.e., global best). Based on the particles velocity, position of the particle is updated using the following expression:

(13) $$\begin{array}{c} p_{n}^{k}=p_{n}^{k-1}+v_{n}^{k}. \end{array}$$

In essence, once the swarms are initialized (particles initial positions, random speed vector for each particle, and setting of the *g*_best_ and *p*_best_), an updating procedure is required in every next iteration. Equations (12) and (13) are used to update the speed and velocity of the particle, while the fitness of the particles is calculated according to (10). The images are ranked from lower to higher fitness, and then relevant repository is updated. This process is terminated when any of these conditions is met: (1) total numbers of intended iterations are completed and (2) system retrieved the predefined number of relevant images. The output generated from the PSO is used to train the SVM which will classify the relevant and irrelevant images. Finally, the relevant images are displayed to the user.

## 4. Experimental Setup

This section discusses the assessment and validation of the proposed PSO-SVM-RF. Assessment is aimed to check performance of the system carried out through experiments on real data set. The validation process involves the comparison of the results obtained during the assessment process of PSO-SVM-RF with other well-known RF based CBIR techniques. The details of real data set and experiments performed are discussed in the following sections.

### 4.1. Image Database and Performance Metrics

To perform experiments, this study used the Coral database [35] consisting of two versions as Coral set A and Coral set B. Coral set A has 1000 images divided into 10 classes where each class contains 100 images. The classes are Elephants, Africa, Beach, Buses, Buildings, Flowers, Dinosaurs, Mountains, Food, and Horses. Corel set B has 9908 images divided into several categories like sunset, texture, butterfly, birds, animals, jungle, cars, boats, and so forth. In this study, both versions of the data sets are merged together into a single image repository containing 10908 images so that each class can have at least 100 images and all the classes are homogeneously categorized.

Experiments are performed by running the simulation using MATLAB 2010b for 300 images, which are selected randomly as query images. Similar images against each query image are displayed to user based on the distance between the query image and the database images. RF is executed automatically and all the images from displayed results which are similar in semantic with query image are marked as positive samples while rest of the images are marked as negative samples. To check the robustness of PSO-SVM-RF, these experiments are performed for different top retrievals ranging from top 10 to top 100. Top retrievals are the number of similar images desired by user to display. For example; if the user wants to display 10 images it means top retrieval is 10 and if user is interested in showing the 20 most similar images then top retrieval is 20. In each experiment RF is repeated 9 times.

Performance of the PSO-SVM-RF is measured using precision and recall. Precision and recall are calculated using following expressions:

(14) $$\begin{array}{c} \text{Precision}=\frac{\text{Number of relevant images}}{\text{Total Number of retrieved images}},\\ \text{Recall}=\frac{\text{Total number of relevant retrieved images}}{\text{Total number of relevant images in the database}}. \end{array}$$

The evaluation of the PSO-SVM-RF depends upon the precision value which ranges from 0.1 to 1.0. Typically, the higher the precision value the higher the performance of the system and vice versa. Averaged precision value of all 300 queries for each iteration is presented in a graph called precision curve. The precision curve evaluates the effectiveness of PSO-SVM-RF. On the other hand, the robustness of the PSO-SVM-RF is evaluated based on the recall value (Figure 4). It is very important to note that in case of less top retrieval the precision is high and recall is low, but when the number of top retrieval is increased, the precision decreases and recall increases.

### 4.2. Visual Signature

Significant set of visual features such as colors, textures, contours, and shapes [36, 37] is very important for any CBIR system. Although in-depth analysis of image is not the objective of this paper, but to best describe the image, homogeneous texture descriptor (HTD) [34] from the MPEG-7 descriptors is used in this study. The reasons to use HTD are because it is fast and successful in texture representation and captures global features as described by Xu and Zhang [38].

The feature extraction was performed offline. The extracted feature vectors representing each image were stored in a repository for run time access. As mentioned above, the size of each feature vector is 62, where 30 features are energy values of each channel, 30 features are the energy deviation of each channel, 1 is the mean, and 1 is the standard deviation of the image.

### 4.3. Analysis

**Figure 3:**
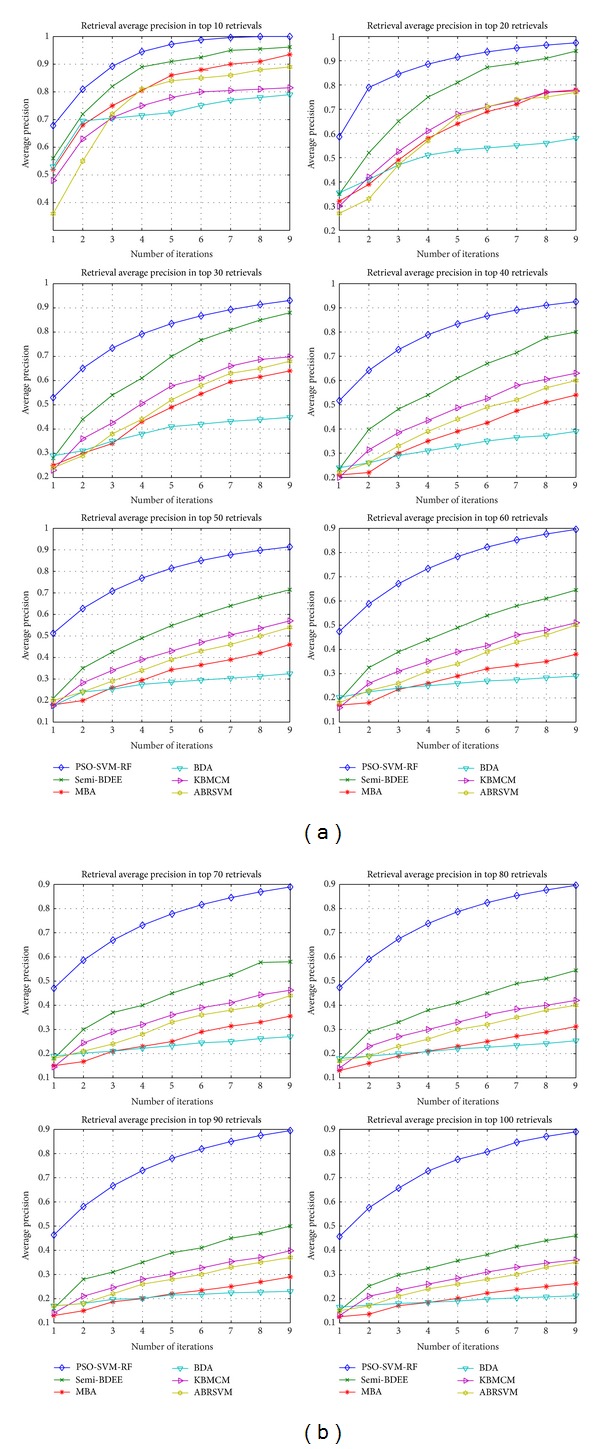
Performance of the proposed PSO-SVM-RF compared against the representative of existing algorithms, that is, semi-BDEE, MBA, BDA, KBMCM, and ABRSVM. All algorithms are evaluated over nine RF iterations.

**Figure 4:**
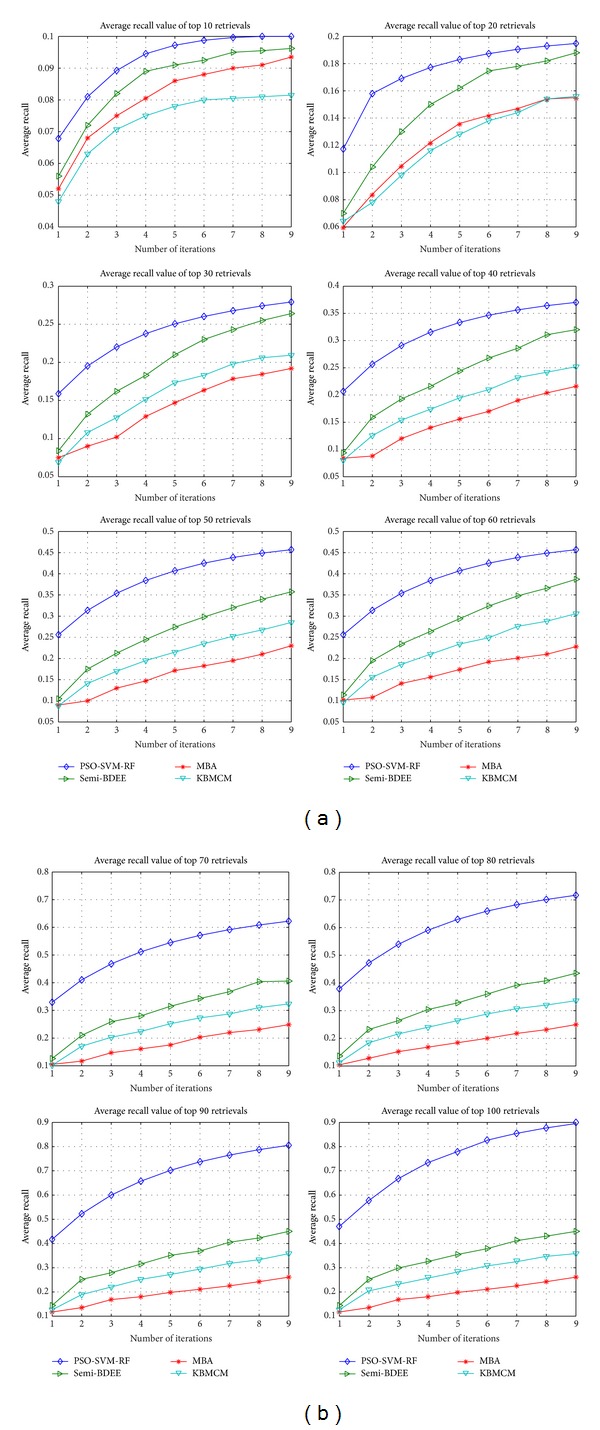
Recall based comparison of the proposed PSO-SVM-RF against representative of existing algorithms, that is, semi-BDEE, MBA, BDA, KBMCM, and ABRSVM. All algorithms are evaluated over nine RF iterations.

**Figure 5:**
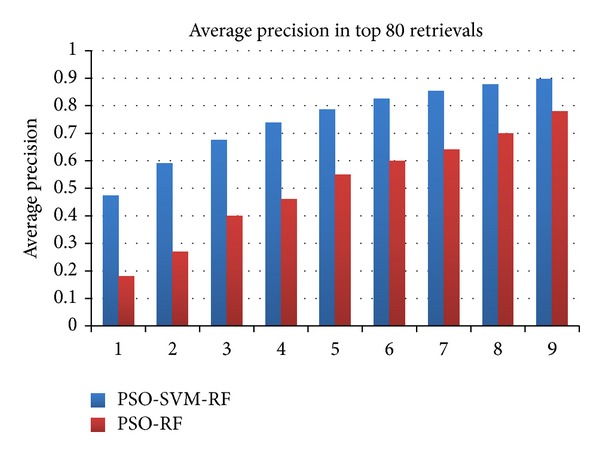
Performance of the proposed PSO-SVM-RF compared against PSORF [33].

The performance of PSO-SVM-RF is compared with the semi-BDEE [39], DBA [40], ABRSVM [13], MBA [41], and kernel biased marginal convex machine (KBMCM) [1]. For comparison purpose the results of the above-mentioned CBIR techniques with similar experimental setup as of PSO-SVM-RF are adopted from Bian and Tao [39] as shown in Figure 3. From Figure 3, it is perceived that average precision of PSO-SVM-RF is similar to semi-BDEE for top 10, 20, and 30 retrievals and is better than the other techniques. For top 40 retrievals, precision is achieved higher than 0.9 which is higher than all other techniques. In case of 50, 60, 70, 80, 90, and 100 top retrievals the average precision achieved by PSO-SVM-RF is 0.9 while the maximum average precision by other techniques is 0.71, 0.64, 0.59, 0.55, 0.5, and 0.48, respectively. These results highlight that PSO-SVM-RF has consistently outperformed semi-BDEE, MBA, BDA, ABRSVM, and KBMCM in all iterations and all top retrievals. It has promising convergence in earlier iterations compared to all other techniques, which means that the user can achieve desired results in few iterations only. Furthermore, PSO-SVM-RF technique is also compared with PSO-RF [33] and comparison graph is presented in Figure 5. From Figure 5 it can be noticed that in each iteration PSO-SVM-RF has higher precision value than PSO-RF precision. This demonstrates that the pairing of SVM with PSO has made RF more robust and enhanced the accuracy of CBIR. Hence it can be concluded that the developed PSO-SVM-RF technique is a precise and efficient CBIR technique.

## 5. Conclusion

This paper has introduced a PSO based SVM approach to enhance the performance of CBIR. It incorporated PSO to boost the number of relevant images compared to the user selected positive images. This resulted in providing large training set for SVM with higher number of relevant images in positive sample. There is no specific limit to the training data as it depends on the user input and then the stochastic process of PSO. PSO generates two sets of images, relevant and irrelevant, which are used for the training of SVM. With this, the problem of overfitting of SVM is reduced. PSO-SVM-RF successfully reduced the semantic gap between low level features and high level semantic. In the developed PSO-SVM-RF approach, the SVM is trained on the best particle among the search space where best particle is searched by PSO algorithm. Every particle represents an image in the database. Experimental work was carried out using Corel photo database with 10,908 images and the results were compared with several popular RF algorithms, like semi-biased discriminant Euclidean embedding (semi-BDEE), marginal biased analysis (MBA), ABRSVM, biased discriminant analysis (BDA), and kernel biased marginal convex machine (KBMCM). It was found that PSO-SVM-RF improved the performance of RF significantly. It achieved 100% accuracy in the 8th feedback iteration for retrieving 10 most similar images while accuracy was higher than 90% for the retrieving of 20, 30, and 40 most relevant images. In case of retrieving 50–100 most similar images the accuracy was achieved approximately 90%, which shows that the proposed technique is robust, stable, and useful in reducing the number of feedback.
